# GSK-3 Inhibition Modulates Metalloproteases in a Model of Lung Inflammation and Fibrosis

**DOI:** 10.3389/fmolb.2021.633054

**Published:** 2021-06-21

**Authors:** Francesco Cinetto, Jessica Ceccato, Ilaria Caputo, Daniela Cangiano, Barbara Montini, Francesca Lunardi, Maria Piazza, Carlo Agostini, Fiorella Calabrese, Gianpietro Semenzato, Marcello Rattazzi, Carmela Gurrieri, Riccardo Scarpa, Carla Felice, Fabrizio Vianello

**Affiliations:** ^1^ Internal Medicine and Allergology and Clinical Immunology Units, Treviso Ca’ Foncello Hospital, Treviso, Italy; ^2^ Hematology Unit, Department of Medicine, University of Padova, Padova, Italy; ^3^ Institute of Pediatric Research (IRP) Città Della Speranza, Padua, Italy; ^4^ Department of Cardiothoracic and Vascular Sciences, Pathology Section, University of Padova, Padua, Italy

**Keywords:** idiopathic lung fibrosis, metalloproteases, glycogen synthase kinase 3, bleomycin-induced lung injury, extracellular matrix (ECM)

## Abstract

Idiopathic pulmonary fibrosis (IPF) is mainly characterized by aberrant extracellular matrix deposition, consequent to epithelial lung injury and myofibroblast activation, and inflammatory response. Glycogen synthase kinase 3 (GSK-3) is a serine–threonine kinase involved in several pathways, and its inhibition has been already suggested as a therapeutic strategy for IPF patients. There is evidence that GSK-3 is able to induce matrix metalloproteinase (MMP) expression and that its inhibition modulates MMP expression in the tissues. The aim of our study was to investigate the role of GSK-3 and its inhibition in the modulation of MMP-9 and -2 in an *in vivo* mouse model of lung fibrosis and *in vitro* using different cell lines exposed to pro-inflammatory or pro-fibrotic stimuli. We found that GSK-3 inhibition down-modulates gene expression and protein levels of MMP-9, MMP-2, and their inhibitors TIMP-1 and TIMP-2 in inflammatory cells harvested from bronchoalveolar lavage fluid (BALF) of mice treated with bleomycin as well as in interstitial alveolar macrophages and cuboidalized epithelial alveolar cells. To the same extent, GSK-3 inhibition blunted the increased MMP-9 and MMP-2 activity induced by pro-fibrotic stimuli in a human lung fibroblast cell line. Moreover, the αSMA protein level, a marker of fibroblast-to-myofibroblast transition involved in fibrosis, was decreased in primary fibroblasts treated with TGFβ following GSK-3 inhibition. Our results confirm the implication of GSK-3 in lung inflammation and fibrosis, suggesting that it might play its role by modulating MMP expression and activity but also pushing fibroblasts toward a myofibroblast phenotype and therefore enhancing extracellular matrix deposition. Thus, its inhibition could represent a possible therapeutic strategy.

## Introduction

Idiopathic pulmonary fibrosis (IPF) is characterized by extensive lung parenchyma remodeling due to the abnormal deposition of extracellular matrix (ECM) by fibroblasts and the migration of epithelial cells and myofibroblasts through the disrupted basement membrane (BM) into the alveolar spaces (Selman et al., 2001).

In this context, matrix metalloproteinases (MMPs), a family of extracellular and zinc-dependent enzymes, are proposed to play a crucial role through their proteolytic activity (Woessner, 1991).

MMP activity is regulated at multiple levels including gene transcription and extracellular activation of the zymogen and inactivation by specific inhibitors referred to as tissue inhibitors of metalloproteinases (TIMPs) (Chakraborti et al., 2003). Accumulating evidence indicates that an imbalance between MMPs and TIMPs may lead to the alteration of ECM metabolism in a variety of pulmonary disorders, including IPF, emphysema, asthma, and lung carcinoma (Urbanski et al., 1992; Hayashi et al., 1996; Betsuyaku et al., 1999; Selman et al., 2000; Suga et al., 2000; Ramos et al., 2001; Russell et al., 2002; Kelly and Jarjour, 2003).

Two gelatinases, MMP-9 (gelatinase B) and MMP-2 (gelatinase A), are of particular interest as they are able to degrade the common substrates collagen type IV, the major constituent of the BM, and gelatin. These two gelatinases greatly differ in transcription control, with MMP-2 constitutively expressed while MMP-9 being induced by soluble factors such as cytokines and growth factors and by integrin-mediated signaling through cell–matrix or cell–cell interactions (He, 1996; Hrabec et al., 2002; Chakraborti et al., 2003). Evidence suggests that inducible MMP-9 may have multiple roles in the lung, with studies implicating it in wound repair of human respiratory epithelium as well as in pathological processes including alveolar bronchiolization in bleomycin-induced lung injury (Buisson et al., 1996; Aoudjit et al., 1998; Legrand et al., 1999; Lemjabbar et al., 1999).

We previously identified anti-inflammatory and anti-fibrotic properties of the specific inhibitor of glycogen synthase kinase 3 (GSK-3), SB216763, in a mouse model of bleomycin (BLM)-induced lung inflammation and fibrosis (Gurrieri et al., 2010). GSK-3, a pleiotropic serine–threonine kinase, is known as a crucial mediator of inflammation homeostasis and is implicated in pathways controlling cell proliferation and survival. It is constitutively active and inhibited, rather than activated, in response to stimulation of two main signaling pathways, insulin and Wnt/β-catenin pathways.

It has been shown that GSK-3β is an inducer of MMP-9 expression through the activation of NF-*κ*B and that MMP-9 can be down-modulated by GSK-3 inhibition (Labrie and St-Pierre, 2013; Rocha et al., 2015). To the same extent, GSK-3β inhibition attenuates the invasion of collagen gel by several types of cancer cells by decreasing the secretion and activity of MMP-2 (Kitano et al., 2013; Chikano et al., 2015; Abe et al., 2020). GSK-3 is also involved in TGFβ-1–dependent differentiation to myofibroblasts and in epithelial-to-mesenchymal transition (Baarsma et al., 2013; Caraci et al., 2008; Kim et al., 2014).

In this study, we aim to investigate the *in vivo* and *in vitro* roles of GSK-3 inhibition in the modulation of MMP-9 and MMP-2 and of their inhibitors TIMP-1 and TIMP-2 in the development of lung fibrosis.

## Materials and Methods

### Mice

C57BL/6N mice obtained from Charles River Laboratories and The Jackson Laboratory, Inc. (Milan, Italy), were used in this study. Mice were housed under ethical conditions in a pathogen-free animal facility. Mice were used at 12 weeks of age. All procedures were approved by the local Animal Care Committee of the University of Padova (Padova, Italy).

### Experimental Protocol

We used the maleimide SB216763 as a selective ATP-competitive GSK-3 inhibitor (Coghlan et al., 2000). C57BL/6N mice were randomized into four different subgroups (*n* = 15/group), and they received saline, saline plus SB216763 (control groups), BLM plus vehicle, and BLM plus SB216763. As previously shown and confirmed in our experiments (not shown), no significant differences were detected at any level between saline and saline plus SB216763 groups, and therefore, when mentioning control in the text, we refer to saline only (Liu et al., 2013). Mice were anesthetized and treated with intratracheal administration of isotonic saline or bleomycin sulfate (3 U/kg) (Aventis Pharma SpA, Varese, Italy) as previously described. SB216763 (20 mg/kg) (Sigma-Aldrich, St. Louis, MO) dissolved in dimethyl sulfoxide and polyethylene glycol was administered intraperitoneally twice a week, as previously described (Gurrieri et al., 2010). Mice underwent bronchoalveolar lavage (BAL) and were euthanized 7 days after BLM or saline administration, and lungs were then processed as previously described (Gurrieri et al., 2010).

### Histologic Examination and Histochemistry

Lung tissues were formalin-fixed and paraffin-embedded, and 4–5 *μ*m sections were stained with hematoxylin and eosin (H&E), to evaluate the degree of inflammatory cell infiltration and alveolar epithelial cuboidalization, and stained with Masson’s trichrome to evaluate the degree of interstitial fibrosis. Then, each section was scanned at 40x magnification to identify at least five areas (hot spots) with the largest extension of fibrosis (trichrome staining). Each hot spot was then examined at x200 magnification (0.949 mm^2^/field), and fibrosis was quantified by using digital quantitative analysis (Image-Pro Plus software version 4.1, Media Cybernetics, Silver Spring MD). The mean value of the five areas was taken as representative of the whole section.

For immunohistochemical analyses, following dewaxing and hydration, sections were incubated in citrate buffer 5 mM at pH 6.0 in a microwave oven for 30 min for antigen retrieval. Afterward, sections were treated with blocking serum (Ultratech HRP Kit; Immunotech, Beckman Coulter, United States) and incubated for 60 min with the mouse monoclonal antibodies anti-MMP-2, -MMP-9, -TIMP-1, and -TIMP-2 (Santa Cruz Biotechnology, CA) at concentrations of 1:200, 1:800, 1:200, and 1:500, respectively. Sections were subsequently incubated with a secondary biotinylated antibody for 10 min and then with the streptavidin–biotin complex conjugated to horseradish peroxidase for 10 min (Ultratech HRP Kit; Immunotech, Beckman Coulter). Immunoreactivity was visualized with diaminobenzidine (DAB; Dako, Denmark). Finally, the sections were counterstained with Mayer’s hematoxylin. Negative controls for non-specific binding were processed omitting the primary antibodies and revealed no signal. Immunoreactivity for MMP-9, TIMP-1, MMP-2, and TIMP-2 was evaluated in at least 100 macrophages and metaplastic epithelial cells and expressed as percentage compared with the total amount of nucleated cells. Two experienced pathologists (FC and FL) performed quantification, and the mean value was considered for the statistical analyses.

### Bronchoalveolar Lavage and Cell Count in Bronchoalveolar Lavage Fluid

Airways were lavaged three times with 0.4 ml of sterile saline. BAL was centrifuged and the supernatant was stored at −80°C for zymographic analysis. BAL cells were adjusted to the final concentration of 1 × 10^6^ cells/ml in phosphate buffer saline, and total cell counts were performed by manual counting under light microscopy with a standard hemocytometer chamber. Finally, 100 *μ*l of BAL cells were smeared on a glass slide and then stained with May-Grünwald-Giemsa dyes. Differential counts on 200 cells were made using standard morphological criteria.

### Gelatin Zymography

Equal amounts of secreted proteins (5 *μ*g) from BAL fluid were mixed with 4X non-reducing sample buffer (1.25 M Tris-HCl, pH 6.8, 10% (w/v) sodium dodecyl sulfate (SDS), 40% (v/v) glycerol, 1% bromophenol blue) (3:1, v/v) and electrophoresed on 8% SDS-PAGE containing 1% gelatin (Sigma-Aldrich, St. Louis, MO) as MMP-9 and MMP-2 substrates. Following electrophoresis, the gels were washed twice with 2.5% Triton X-100 and then incubated overnight at 37°C in developing buffer (50 mM Tris-based, 200 mM NaCl, 10 mM CaCl_2_, pH 7.4). The gels were stained with 0.5% (w/v) Coomassie Brilliant Blue R-250 (Sigma-Aldrich, St. Louis, MO) in 30% methanol and 10% acetic acid and destained in a solution of 30% methanol and 10% acetic acid. Gelatinases appear as clear bands against blue background, with recombinant protein molecular weight markers used to identify the weights of the gelatinolytic bands. Relative enzyme amounts were quantified by measuring the intensity of the bands with the pixel-based densitometer program Quantity One^®^ 1-D Analysis Software (Bio-Rad Laboratories, Inc., Hercules, CA). For cell culture studies, cell culture supernatant media were collected and concentrated by Amicon Ultra 3K (Merck Millipore KGaA, Darmstadt, Germany) prior to zymography, and a final volume of 10 *μ*l was loaded for each sample. Densitometry for cell line zymography was performed with Image Lab (Bio-Rad Laboratories, Inc., Hercules, CA).

### Cell Lines and Treatments

MRC5 cells (CCL-171, purchased from ATCC) were cultured in Dulbecco’s modified Eagle’s medium w/L-glutamine (1%), w/sodium pyruvate (1%), w/non-essential amino acids (1%) (Euroclone, Milan, Italy), supplemented with penicillin and streptomycin (1%) (Euroclone, Milan, Italy), and w/10% v/v fetal bovine serum (FBS) (Euroclone, Milan, Italy). The A549 cell line (CCL-185, purchased from ATCC) was cultured in the same conditions as MRC5 cells but w/o sodium pyruvate.

Primary lung fibroblasts were isolated from both IPF and non-IPF patients. Human primary fibroblasts (kindly provided by Donna E. Davies, Brooke Laboratories, University of Southampton, Southampton, United Kingdom) were isolated as previously described (Conforti et al., 2017). All primary fibroblasts were used for experiments between passages 3 and 6. Clinically indicated IPF lung biopsy tissue samples and age-matched non-fibrotic control tissue samples (macroscopically normal lung sampled remote from a cancer site in patients undergoing surgery for early-stage lung cancer) deemed surplus to clinical diagnostic requirements were flash frozen and stored in liquid nitrogen. All IPF samples were from patients subsequently receiving a multidisciplinary diagnosis of IPF according to international consensus guidelines (Raghu et al., 2011).

All human lung experiments were approved by the Southampton and South West Hampshire and the Mid and South Buckinghamshire Local Research Ethics Committees (ref 07/H0607/73), and all subjects gave written informed consent.

Primary monocytes were isolated from healthy buffy coats, exploiting their ability to grow attached to the plate. Briefly, monocytes underwent gradient separation by using Lymphosep (Biowest, Nuaillé, France) before and then Percoll (GE Healthcare Bio-Sciences Ab, Uppsala, Denmark). Cells were then counted and plated in a six-well plate at a density of 2 × 10^6^ cells/well. After 1 h, floating cells were discarded and only attached cells (monocytes) were treated with GM-CSF (Miltenyi Biotec, Bologna, Italy) in order to push monocytes toward an M0-like phenotype (Del Prete et al., 1995). After 7 days, GM-CSF was removed and treatments were performed, as described later.

Proliferative cultures (for every cell line) were incubated at 37°C in a humidified 5% CO_2_ incubator, and subculture was carried out by washing the cell monolayers twice with calcium- and magnesium-free Dulbecco’s phosphate-buffered saline (DPBS) (Euroclone, Milan, Italy), followed by addition of 1X Trypsin/EDTA solution (Gibco, Thermo Fisher, Monza, Italy) and incubation at 37°C until the cells detached. The cells were seeded (1.5–2x10^5^ cells/well) using six-well plates.

Cells (fibroblasts, alveolar epithelial cells, and monocyte/macrophage-derived cells) were starved and treated with TNFα (Sigma-Aldrich, St. Louis, MO) at a concentration of 15 ng/ml or TGFβ (PeproTech, London, United Kingdom) at 2–5 ng/ml, in the presence or absence of SB216763 (Sigma-Aldrich, St. Louis, MO), an inhibitor of GSK-3, used at a concentration of 8 *μ*M for A549 and 10 *µ*M for fibroblasts and macrophages, on the basis of what reported in the literature and of our preliminary data (Baarsma et al., 2013). Experiments were performed for 24 or 48 h in complete serum starvation.

### Western Blot

After 24 and 48 h of stimulation with TNFα or TGFβ, proteins were extracted with tissue protein extraction reagent (Santa Cruz, CA, United States) with the addition of a protease inhibitor (Roche, Basel, Switzerland). The concentration was then measured using the Bradford quantification assay (Pierce, Thermo Scientific, Rockford, IL). Equal amounts of proteins (10 µg) were denatured in Laemmli buffer (Bio-Rad Laboratories, Inc., Hercules, CA) added with β-mercaptoethanol (Sigma-Aldrich, St. Louis, MO). Samples were boiled for 4 min, separated by 10% SDS-PAGE gel, and electrophoretically transferred onto PVDF membranes (Thermo Scientific, Rockford, IL). The membranes were blocked for 1 h at room temperature with 5% non-fat dry milk in TBS added with 0.1% Tween-20 (Sigma-Aldrich, St. Louis, MO), followed by overnight incubation at 4°C with the following antibodies: MMP-9 (Merck Millipore KGaA, Darmstadt, Germany); MMP-2 (Merck Millipore KGaA, Darmstadt, Germany); TIMP-1 (Abcam, Cambridge, United States); TIMP-2 (Abcam, Cambridge, United States); αSMA (Sigma-Aldrich, St. Louis, MO); and GAPDH (Merck Millipore KGaA, Darmstadt, Germany).

### Real-Time PCR Amplification

mRNA was extracted from BALF inflammatory cells using TRIzol reagent (Invitrogen Life Technologies, Grand Island, NY) and 1 *μ*g of RNA reverse transcribed into cDNA using Reverse Transcription System (PROMEGA, Madison, WI) according to the manufacturer’s instructions. Real-time PCR amplification was performed on 100 ng of cDNA on an ABI PRISM 7000 sequence detection system (Applied Biosystems, Foster City, CA). Reactions were carried out with the Platinum^®^ SYBR^®^ Green qPCR SuperMix-UDG kit (Invitrogen Life Technologies, Carlsbad, CA) at an annealing temperature of 58°C. The primer sequences are reported in Table 1. β-Actin was used as a housekeeping gene. Data were first calculated as the mean of the ratio of the target mRNA to β-actin and subsequently normalized to the control group.

**TABLE 1:** Primers used for quantitative real-time PCR.

Gene	Forward primer (3′–5′)	Reverse primer (3′–5′)	Amplicon length (bp)
β-Actin	CTC TCC CTC ACG CCA TCC TG	TCA CGC ACG ATT TCC CTC TCA G	269
MMP-9	CGA CGG CAA GGA CGG C	GTA AGT GGG GAT CAC GAC GC	129
MMP-2	CGG TTT ATT TGG CGG ACA GTG AC	ATT CCC TGC GAA CAC AGC	144
TIMP-1	TGG CAT CCT CTT GTT GCT ATC ACT G	TGA ATT TAG CCC TTA TGA CCA GGT CC	170
TIMP-2	TGC AGA CGT AGT GAT CAG AGC CAA A	AAC TCG ATG TCT TTG TCA GGT CCT T	144

### Statistical Analysis

All data are expressed as mean ± standard deviation (SD). Statistical differences among groups were determined using Student’s *t*-test. Significance was defined at *p* < 0.05. Analysis and graphs were realized using GraphPad Prism 7.0 (GraphPad Software, Inc., San Diego, CA). Multiple comparisons were performed using one-way ANOVA followed by Tukey’s test. Statistically significant differences were defined at *p* < 0.05.

## Results

### Bronchoalveolar Lavage Fluid Cell Composition

We previously demonstrated that the intratracheal administration of BLM induced pulmonary alveolitis peaking at day 7, and these results have been confirmed in this study (Supplementary Table S1) (Gurrieri et al., 2010).

### Bleomycin-Induced Matrix Metallopeptidase 9 and Matrix Metallopeptidase 2 Activity Is Modulated Following Glycogen Synthase Kinase 3 Inhibition

**FIGURE 1:**
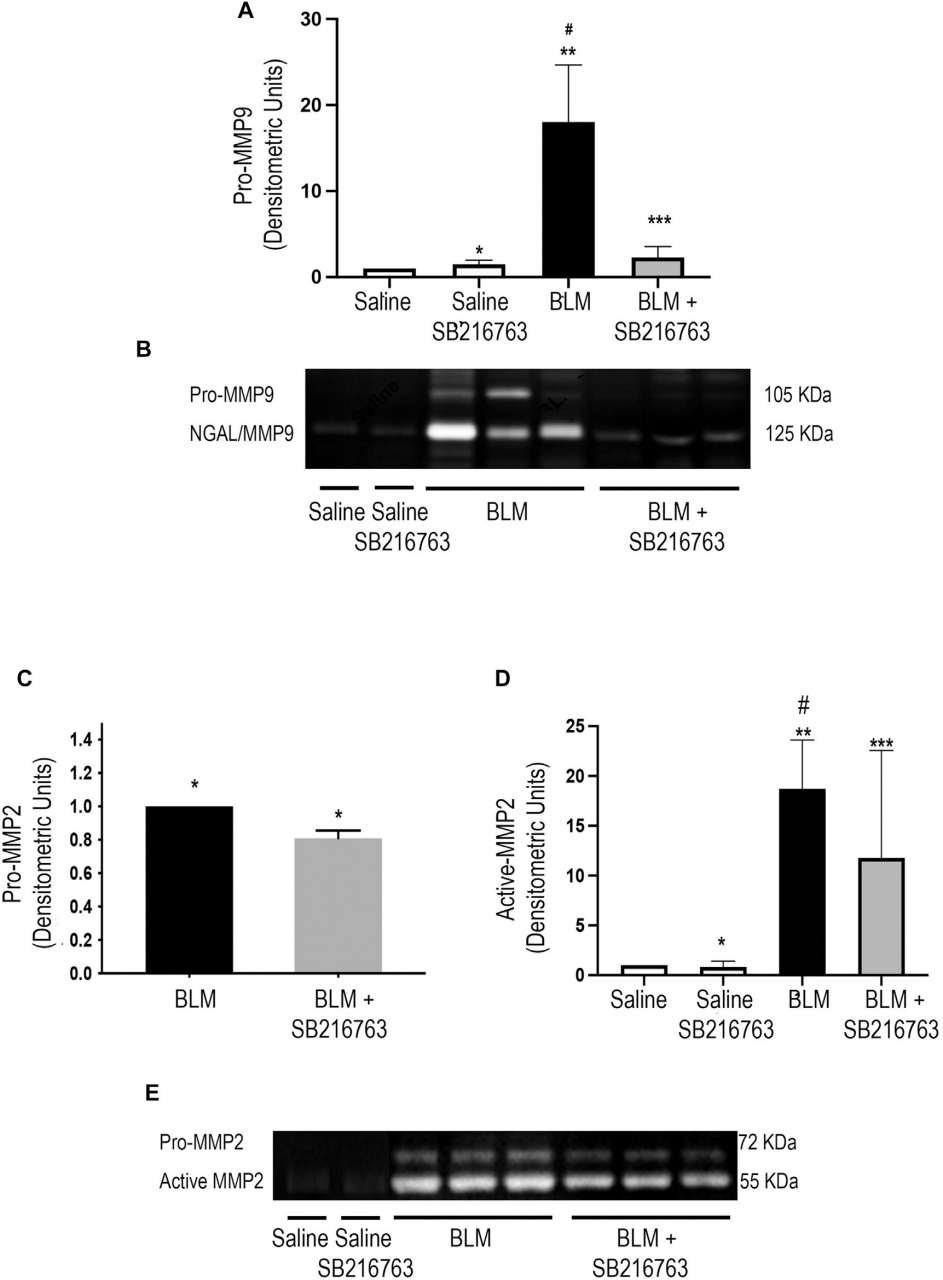
GSK-3 inhibition modulates MMP-2 and MMP-9 activity. **(A)** Densitometric analysis of the intensity of the gelatinolytic bands demonstrated that there was a significant pro-MMP-9 down-modulation by SB216763. Data are given as the mean ±SD of three independent animal trials (*n* = 5/treatment) and were normalized to saline values comparing BLM plus SB216763–treated mice with mice given only BLM. *p = n.s., for saline vs. saline + SB216763; **p < 0.0001 for saline vs. BLM; ***p = n.s. for saline vs. BLM + SB216763; #*p* = 0.0001 for BLM vs. BLM + SB216763. **(B)** Representative gelatin zymography of MMP-9 activity in the BALF of mice from each experimental group. BLM-treated mice had two gelatinolytic bands corresponding to the NGAL/MMP-9 complex (125 kDa) and pro-MMP-9 (105 kDa). The treatment with SB216763 broke down the NGAL/MMP-9 complex and strongly reduced pro-MMP-9. **(C,D)** Densitometric analysis of three independent experiments (*n* = 5/treatment) for active MMP-2 and pro-MMP-2. Protein quantitation for each BALF supernatant was performed, and an equal amount of protein (5 ug) was loaded. Data were normalized to saline values only for active MMP-2 as the pro-MMP-2 signal was undetectable. **(Panel C)** *p = 0.002. **(Panel D)** *p = n.s. for saline vs. saline + SB216763; **p < 0.0001 for saline vs. BLM; ***p = 0.047 for saline vs. BLM + SB216763; #*p* = 0.039 for BLM vs. BLM + SB216763. **(E)** Representative gelatin zymography of MMP-2 activity in BALFs. We detected two gelatinolytic bands corresponding to pro-MMP-2 (72 kDa) and active MMP-2 (52 kDa). Statistical analysis was performed by one-way ANOVA followed by Tukey’s test.

We next performed gelatin zymography to detect MMP-9 and MMP-2 gelatinolytic activity of the BALF supernatant. Control mice (saline or saline plus SB216763) showed very low gelatinolytic activity. In contrast, the instillation of mice with BLM increased MMP-9 activity at day 7, and the zymographic analysis showed two distinct bands at 105 kDa and 125 kDa corresponding to pro-MMP-9 and -MMP-9/neutrophil gelatinase–associated lipocalin complex (NGAL), respectively (Figure 1B). Moreover, SB216763 treatment of mice exposed to BLM broke down the NGAL/MMP-9 complex and strongly reduced the latent form of MMP-9 (Figure 1B). The densitometric analysis indicated that pro-MMP-9 levels of SB216763-treated mice decreased 10 times compared to those of mice given only BLM (*p* < 0.001) (Figure 1A).

Zymographic analysis of MMP-2 revealed two gelatinolytic bands corresponding to the active (52 kDa) and the latent (72 kDa) form in mice treated with BLM (Figure 1E). In this setting, the GSK-3 inhibitor showed a lower but significant modulation of MMP-2 activity, with reduction for active MMP-2 (*p* = 0.039) and pro-MMP-2 (*p* = 0.002) compared to those of BLM-treated mice (Figures 1C–E). TIMP-1 and TIMP-2 were also evaluated by WB analysis in BALF supernatants. We found that bleomycin significantly increased both TIMP-1 and TIMP-2 and that GSK-3 inhibition restored baseline levels (*p* = 0.0003 and *p* = 0.015, respectively) (Supplementary Figure S1).

### Gene Expression Analysis of Matrix Metallopeptidase 9 and Matrix Metallopeptidase 2 in Bronchoalveolar Lavage Fluid Cells

**FIGURE 2:**
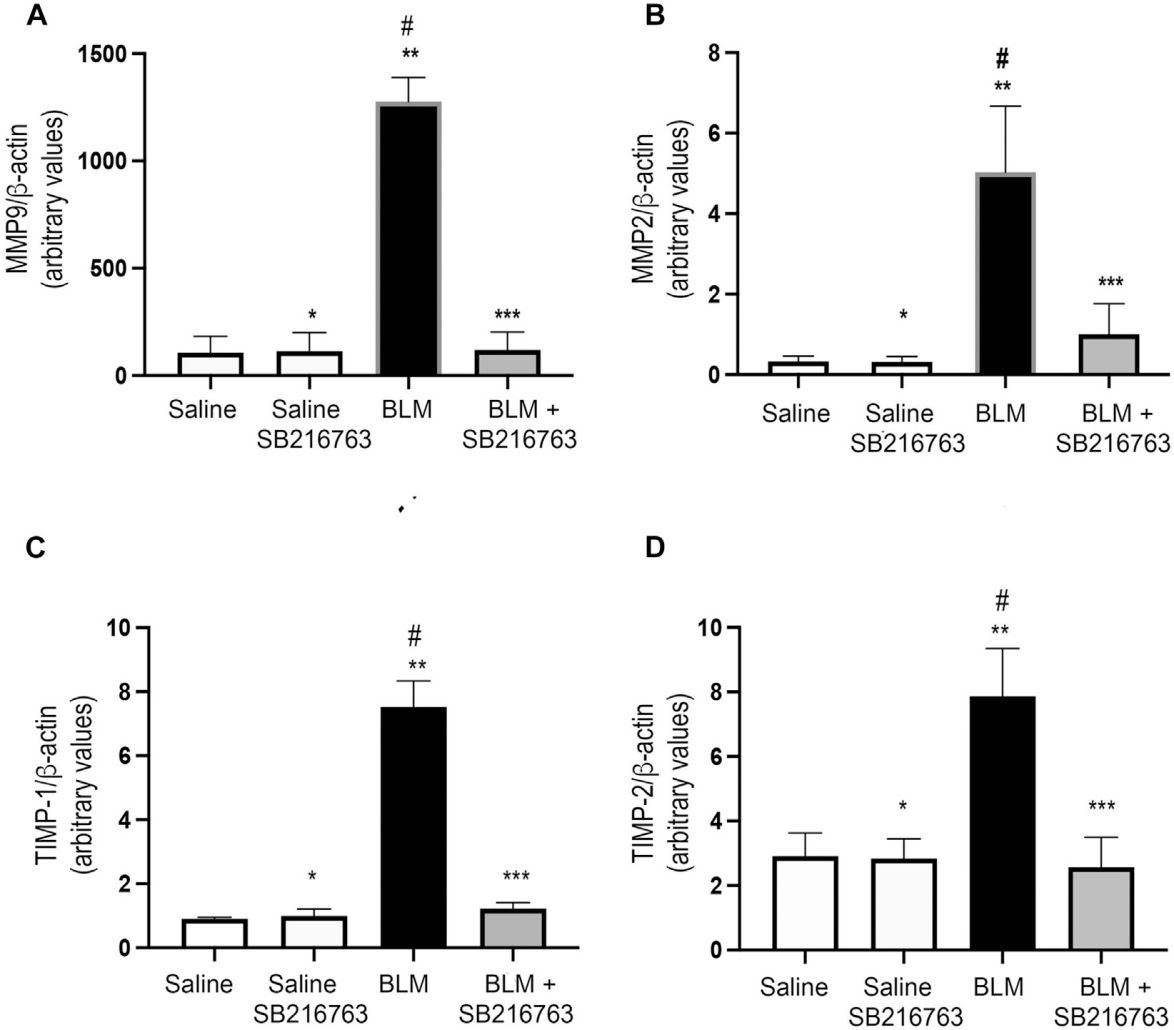
SB216763 reduced MMP-9, MMP-2, TIMP-1, and TIMP-2 gene overexpressions induced by BLM. Real-time PCR analysis of MMP-9 **(A)**, MMP-2 **(B)**, TIMP-1 **(C)**, and TIMP-2 **(D)** mRNAs extracted from the inflammatory cells of the BALFs. **(A)** MMP-9 gene expression was strongly induced in BLM-treated mice, and SB216763 returned it to the normal levels of the control group. **(B)** MMP-2 expression was augmented in mice following BLM instillation, and the co-treatment with SB216763 reduced it. **(C)** TIMP-1 expression was induced by BLM treatment, and SB216763 reduced it to the levels of normal controls. **(D)** SB216763 reduced TIMP-2 mRNA levels increased by BLM treatment. Data, presented as the *fold increase*, represent the mean of the ratio of the target mRNA to β-actin normalized to the control group (saline) and are expressed as the mean ± SD of three independent animal trials. Statistical analysis was performed by Student’s *t*-test or one-way ANOVA followed by Tukey’s test. *p = n.s. for saline vs. saline + SB216763; **p < 0.0001 for saline vs. BLM; ***p = n.s. for saline vs. BLM + SB216763; #*p* < 0.0001 for BLM vs. BLM + SB216763.

Next, we quantified MMP-9 and MMP-2 transcript levels in the cells recovered from BALFs of mice. Mice instilled with BLM showed a very strong increase in MMP-9 gene expression compared to control mice (*p* < 0.0001). The co-treatment of the mice with BLM plus SB216763 reduced MMP-9 mRNA levels to the normal levels of the control group (*p* < 0.0001) (Figure 2A). Similarly, we observed the increase in MMP-2 gene expression in BLM-treated mice compared to control mice (*p* < 0.0001) and that SB216763 co-administered with BLM reduced the augmented MMP-2 mRNA levels (*p* < 0.0001) (Figure 2B).

Then, we assessed gene expression levels of TIMP-1 and TIMP-2, the physiologic inhibitors of MMP-9 and MMP-2, respectively. Although both transcripts were detected at low levels in inflammatory cells collected from BALFs, we found that TIMP-1 and TIMP-2 expressions were augmented in BLM-treated mice compared to control mice (*p* < 0.0001) and the inhibition of GSK-3 reduced the mRNA levels to the values of the control group (*p* < 0.0001) (Figures 2C,D).

### Glycogen Synthase Kinase 3 Inhibition Down-Modulates Matrix Metallopeptidase 9, Tissue Inhibitor of Metalloproteinases 1, Matrix Metallopeptidase 2, and Tissue Inhibitor of Metalloproteinases 2 Overexpressions Induced by Bleomycin in Interstitial Alveolar Macrophages and Cuboidalized Epithelial Alveolar Cells

**FIGURE 3:**
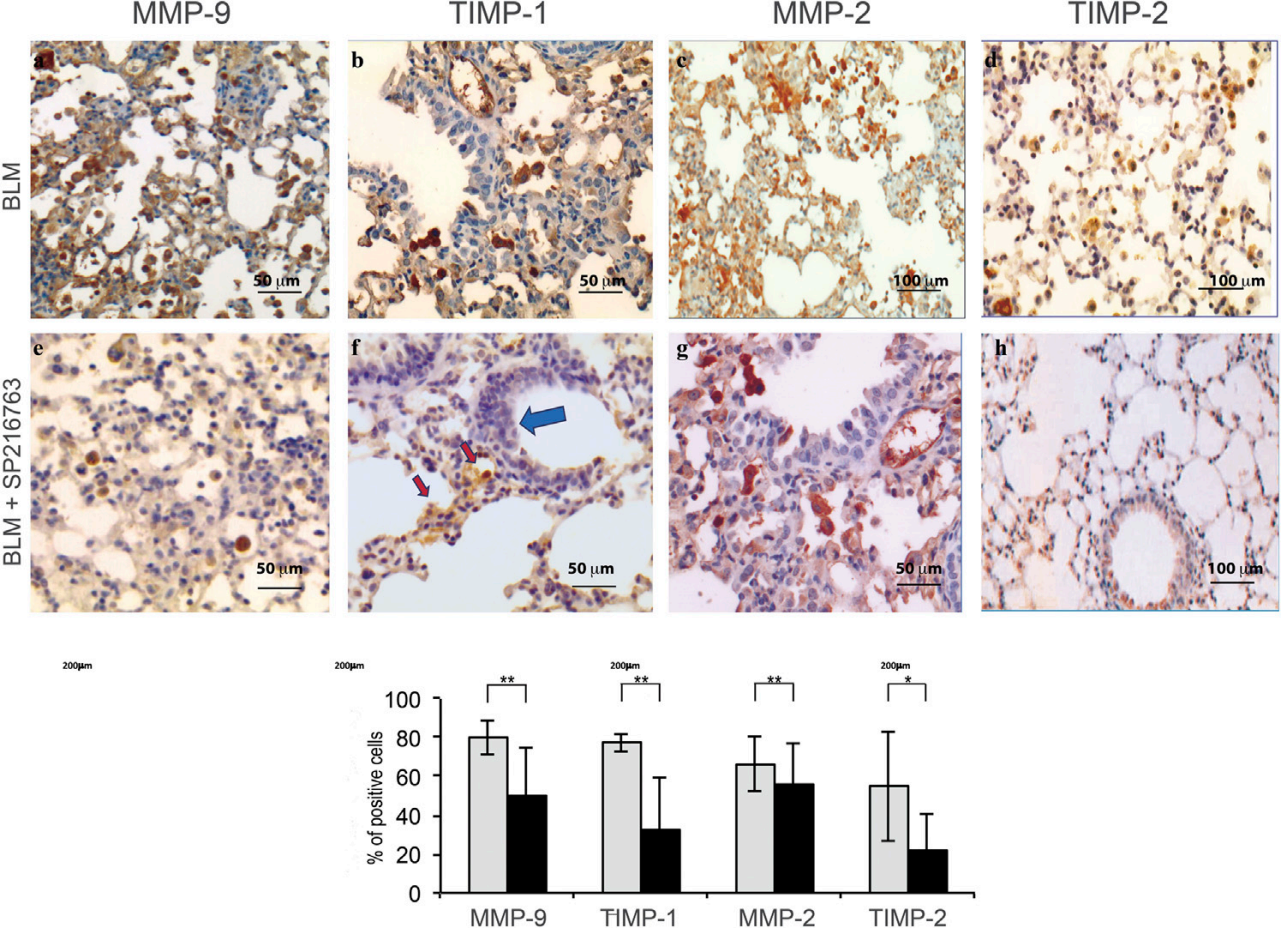
GSK-3 inhibition modulates MMP and TIMP expressions in the lung of mice treated with bleomycin. BLM-treated mice show high positivity for MMP-9, TIMP-1, MMP-2, and TIMP-2 in alveolar macrophages **(Panels A–D)**. GSK-3 administration down-modulates their expression **(Panels E–H)**. Histograms summarize the pathologic scores calculated as the percentage of positive cells for each specific marker. Data are given as the mean ± SD of three independent animal trials. *p < 0.01 and **p < 0.05. Red arrows: interstitial alveolar macrophages; blue arrows: cuboidalized epithelial alveolar cells. Data are representative of three separate experiments (*n* = 15/treatment group).

Immunohistochemistry was performed in all mouse lung samples in order to correlate the tissue expression of MMPs and TIMPs to that observed in BALF. iAMs, the main cell population infiltrating the lung interstitium at this time point, showed a strong staining for MMP-9 (80 ± 8.7%), TIMP-1 (78 ± 4.6%), and MMP-2 (66 ± 14%) and were moderately positive for TIMP-2 (55 ± 28%) at day 7 after BLM instillation (Figure 3). *In vivo* SB216763 co-treatment moderately reduced iAM staining for MMP-9 (50 ± 24%) and markedly down-modulated TIMP-1 (32 ± 26%) and TIMP-2 (22 ± 18%) positivity. MMP-2 expression, instead, was less significantly affected by GSK-3 inhibition (Figure 3).

**FIGURE 4:**
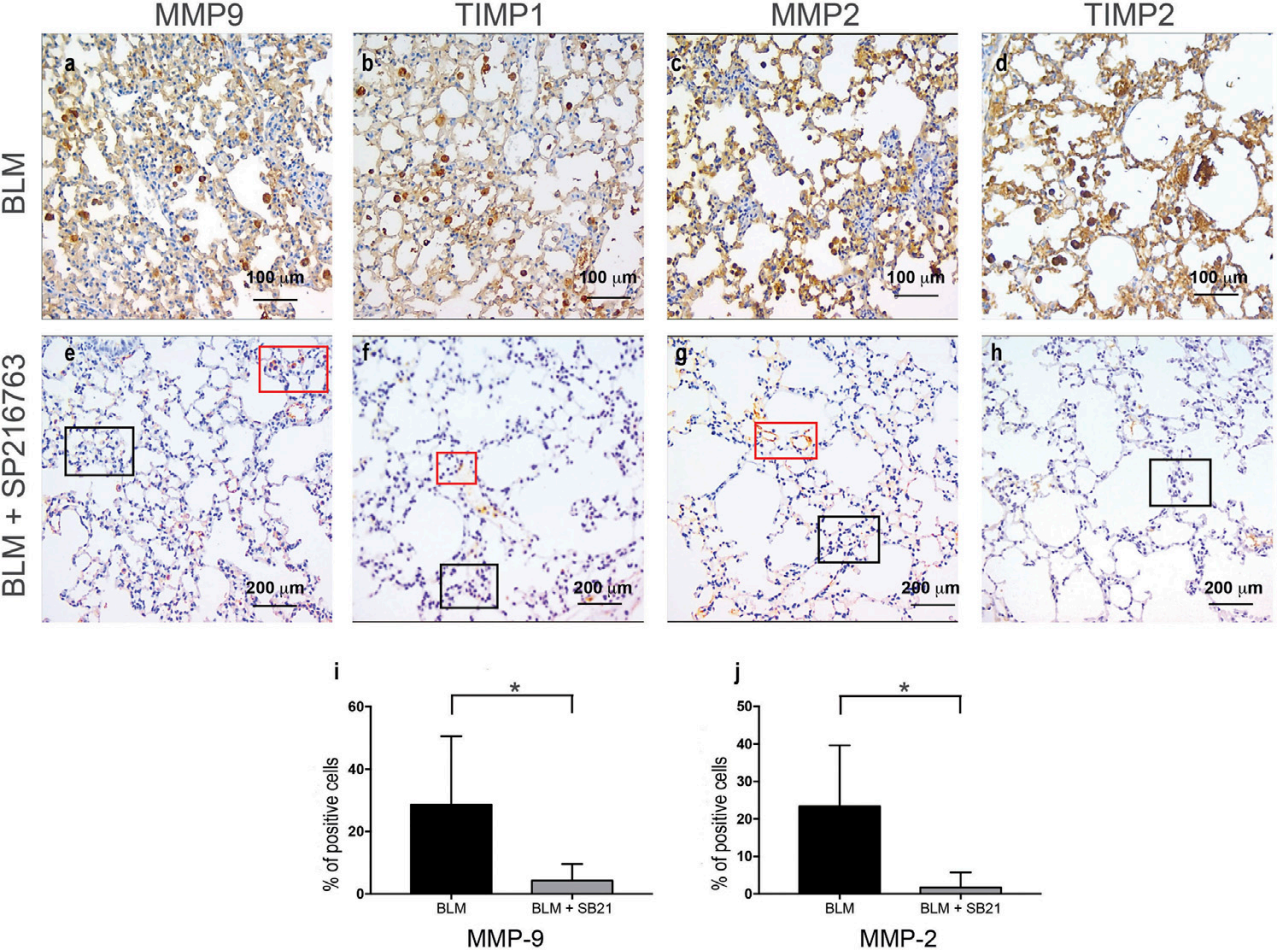
IHC staining of cuboidalized type II epithelial alveolar cells. MMP-9, TIMP-1, MMP-2, and TIMP-2 were specifically expressed in BLM-treated injured alveolar cells that underwent cuboidalization **(Panels A–D)**, and SB216763 selectively reduced their expression **(Panels E–H)**. Histograms summarize the effects of GSK-3 inhibition in reducing BLM-induced epithelial positivity, in particular for MMP-9 **(I)** and MMP-2 **(J)** as shown in the mean ± SD of three independent animal trials by the histogram (*) (*p* < 0.05)*.* Red and blue boxes indicate positive and negative areas, respectively.

When focusing on the metaplastic cuboidalized type II epithelial alveolar cells, a positive staining for MMP-2 (23.3 ± 16.3%), MMP-9 (28.5 ± 22%), TIMP-1 (22 ± 16%), and TIMP-2 (15 ± 17%) was detected after BLM administration. Interestingly, a consistent reduction of MMP-9 and MMP-2 staining in cuboidalized type II epithelial alveolar cells followed co-treatment with SB216763 (from 28.5 to 4.3% and from 23.3 to 1.67%, respectively, at day 7; *p* < 0.05; Figure 4). Moreover, no epithelial staining for TIMP-1 and TIMP-2 was detectable in the SB216763-treated group (Figure 4).

### *In Vitro* Matrix Metallopeptidase Pattern

With the aim of recapitulating the *in vivo* evidence on the role of epithelial alveolar cells and macrophages in MMP and TIMP production, we performed *in vitro* experiments using respective human cell types, namely, epithelial alveolar type II cells (A549), human primary monocytes/macrophages, and human lung fibroblasts (MRC5 cell line and primary IPF fibroblasts).

To prove that SB216763 has activity in our system, we confirmed that the inhibitor is effective in the induction of β-catenin expression in epithelial alveolar type II cells (A549) (data not shown, as known from the literature) (Cross et al., 2001; Wang et al., 2015; Schmid et al., 2017). In our further experiments with A549 cells, GSK-3 inhibition by SB216763 did not significantly affect MMP and TIMP production downstream of TNFα and TGFβ stimulation although both stimuli significantly increased TIMP-1 and TIMP-2 (Supplementary Figure S2).

### Glycogen Synthase Kinase 3 Inhibition by SB216763 Modulates Matrix Metallopeptidase Activity in Pulmonary Fibroblasts

**FIGURE 5:**
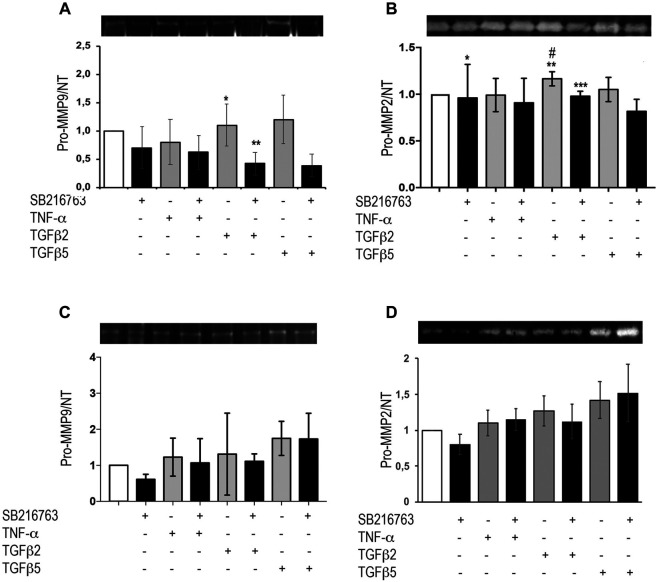
GSK-3 inhibition modulates pro-MMPs *in vitro* in pulmonary fibroblasts. Pro-MMP-2 and pro-MMP-9 zymographic analyses of the supernatant from MRC5 cells **(A,B)** and primary IPF fibroblasts **(C,D)** treated with TNFα or TGFβ. SB216763 pre-treatment induces a decrease in gelatinolytic activity in MRC5 fibroblasts upon pro-fibrotic stimulation (24 h) for pro-MMP-9 **(A)** and pro-MMP-2 **(B)** although TGFβ did not significantly increase pro-MMP-9 compared to control [**(A)** *p = 0.041 for TGFβ2 vs. TGFβ2 + SB216763; **p = 0.033 for saline vs. TGFβ2 + SB216763; **(B)** *p = n.s. for saline vs. saline + SB216763; **p = 0.04 for saline vs. TGFβ2; ***p = n.s. for saline vs. TGFβ2 + SB216763; #*p* = 0.024 for TGFβ2 vs. TGFβ2 + SB216763]. In IPF fibroblasts, instead, SB216763 displays no significant effect on MMP activity **(C,D)**. Data are reported as the mean ± SD of three independent experiments. Statistical analysis was performed by one-way ANOVA followed by Tukey’s test.

MRC5 cells and primary IPF fibroblasts were treated with TNFα or different concentrations of TGFβ as detailed above. Supernatants were collected and underwent zymographic analysis. Our results show that, in MRC5 cells, TGFβ did not significantly increase pro-MMP-9 activity compared to control, but the presence of the GSK-3 inhibitor significantly decreased both the baseline (*p* = 0.033) and TGFβ-induced pro-MMP-9 (*p* = 0.04) (Figure 5A). Moreover, pro-MMP-2 significantly increased following TGFβ (2 ng/ml; *p* = 0.04; Figure 5B), but pre-treatment with SB216763 blunted pro-MMP-2 activity (Figure 5B; *p* = 0.024). No significant differences were observed at 48 h (Supplementary Figure S3). TGFβ and TNFα stimulation of IPF primary fibroblasts did not affect MMP-9 and MMP-2 activity, and no effect of GSK-3 inhibition was observed (Figures 5C,D). No bands corresponding to active MMPs were detected in our experiments with MRC5 and primary IPF fibroblasts.

### SB216763 Decreases α-Smooth Muscle Actin Protein Levels Upon Pro-Fibrotic Stimulation

**FIGURE 6:**
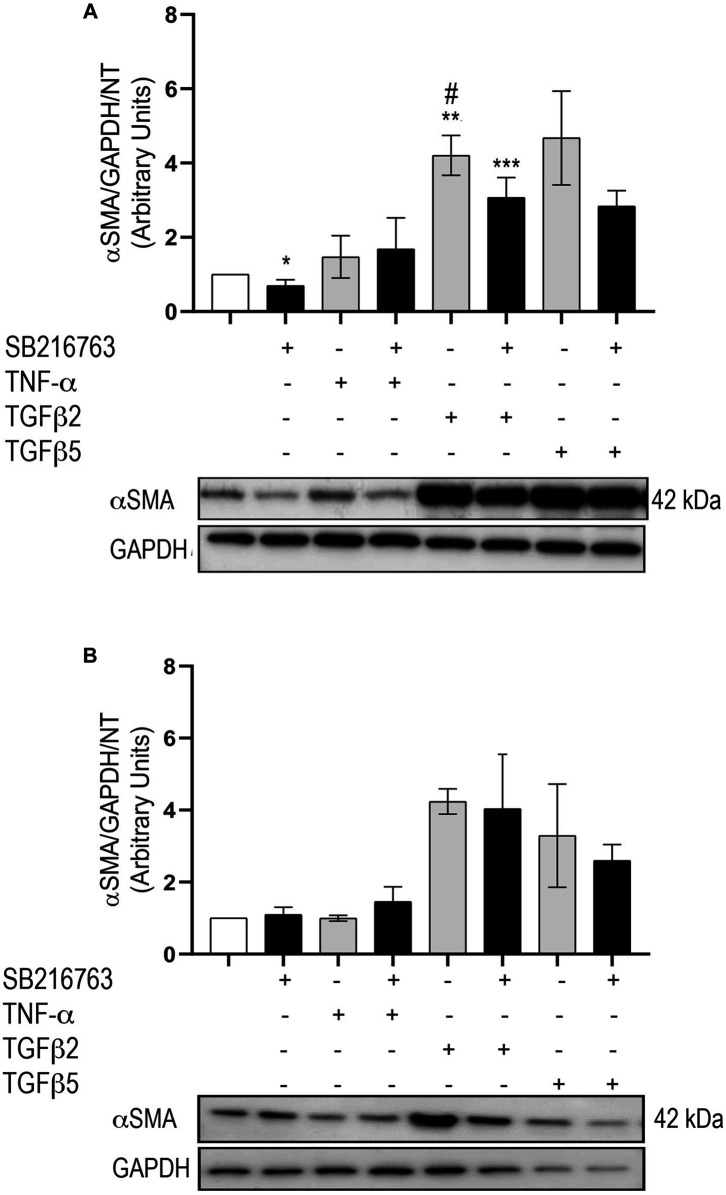
GSK-3 inhibition modulates αSMA expression in primary pulmonary fibroblasts. Western blot analysis of αSMA protein expression in IPF **(A)** and MRC5 **(B)** treated with TNFα or TGFβ. The increased αSMA expression observed with TGFβ is dampened by GSK-3 inhibition. Data are reported as the mean ± SD of three independent experiments. Statistical analysis was performed by one-way ANOVA followed by Tukey’s test *p = n.s. for saline vs. saline + SB216763; **p = 0.008 for saline vs. TGFβ2; ***p = 0.027 for saline vs. TGFβ2 + SB216763; #*p* = 0.016 for TGFβ2 vs. TGFβ2 + SB216763.

In IPF pathogenesis, the differentiation of fibroblasts to myofibroblasts further enhances the ECM aberrant deposition. Therefore, we also studied the expression of αSMA as a marker of this transition using primary IPF fibroblasts and MRC5 cells. The basal expression of αSMA was higher in IPF than MRC5 (not shown). In agreement with published findings (Baarsma et al., 2013), our data confirmed that αSMA protein levels increase downstream of TGFβ stimulation (*p* = 0.008); co-treatment with SB216763 decreased these levels at 48 h, reaching statistical significance at 2 ng/ml TGFβ concentration (*p* = 0.016) in primary fibroblasts (Figure 6A). Consistent with our IPF fibroblast studies, αSMA protein levels in MRC5 cells increased after TGFβ stimulation (2 and 5 ng/ml) and decreased after co-treatment with SB216763, although the decrease did not reach statistical significance (Figure 6B).

### Glycogen Synthase Kinase 3 Inhibition by SB216763 Modulates Matrix Metallopeptidase Protein Expression in Primary Monocytes/Macrophages

**FIGURE 7:**
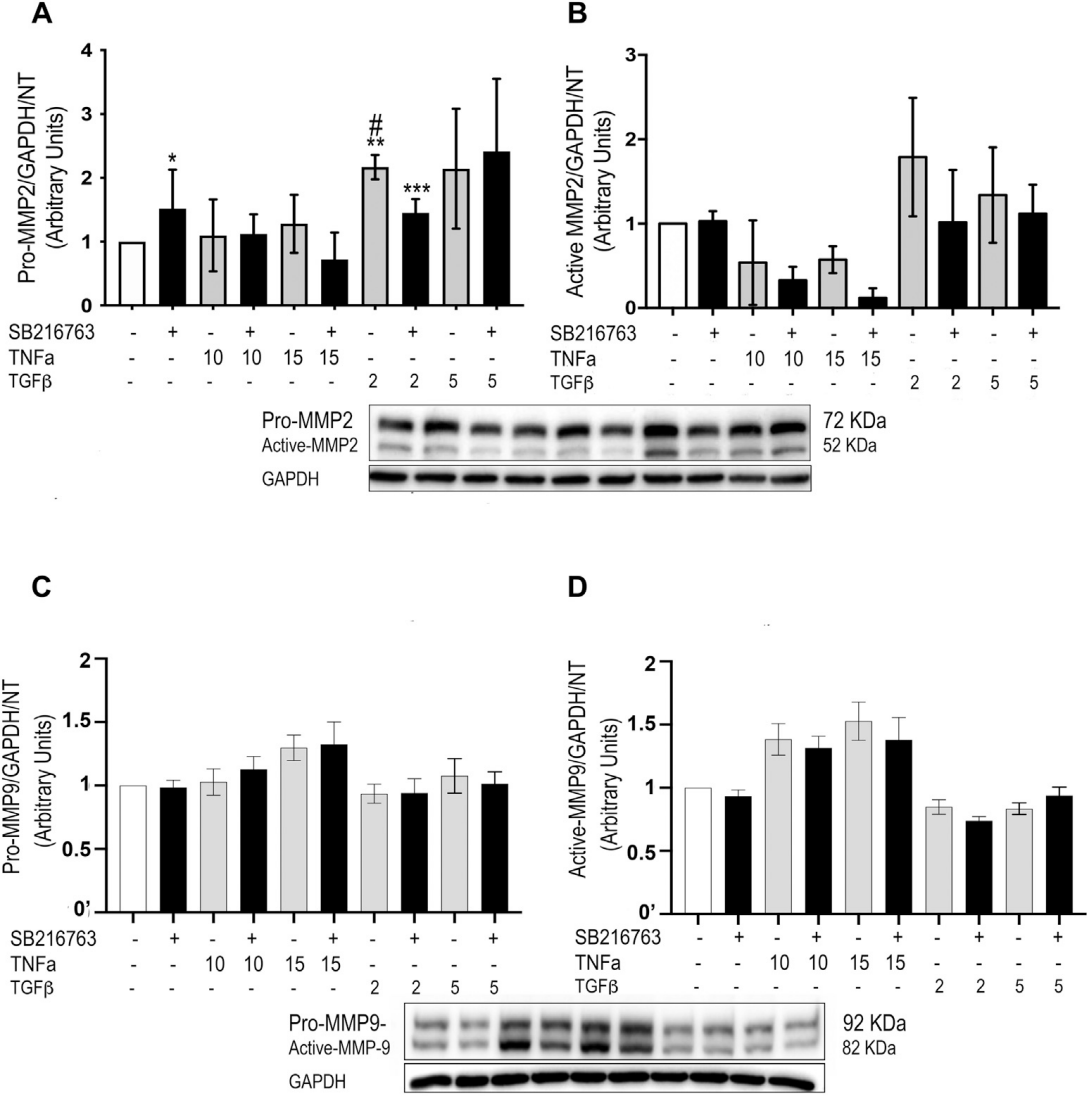
SB216763 modulates MMP-2 protein levels in monocyte-derived cells. WB analysis of MMP-2 and MMP-9 protein levels in primary monocytes/macrophages stimulated with TNFα or TGFβ. Both the TGFβ-induced increased levels of pro-MMP-2 and active MMP-2 proteins are not observed in the presence of the GSK-3 inhibitor at a concentration of 2 ng/ml **(A,B)**. No significant differences are observed for MMP-9 **(C,D)**. Data are reported as the mean ± SD of three independent experiments. Statistical analysis was performed by one-way ANOVA followed by Tukey’s test. *p = n.s. for saline vs. saline + SB216763; **p = 0.043 for saline vs. TGFβ2; ***p = n.s. for saline vs. TGFβ2 + SB216763; #*p* = 0.028 for TGFβ2 vs. TGFβ2+ SB216763.

As we observed a role for macrophages in the inflammatory response in our *in vivo* studies, we then investigated whether GSK-3 inhibition modulated MMP protein expression also *in vitro.* As a model of alveolar macrophages, we generated M0 macrophages from blood monocytes, unpolarized macrophages sharing similarities to the prevalent normal alveolar counterpart (Bazzan et al., 2013; Bazzan et al., 2017). GSK-3 inhibition significantly reduced both pro-MMP-2 and active MMP-2 protein levels triggered by TGFβ stimulation (Figures 7A,B), although the difference reached statistical significance only for pro-MMP-2 (Figure 7A) (*p* = 0.028). No significant differences were observed for pro-MMP-9 and active MMP-9 (Figures 7C,D). Finally, gelatin zymography performed on the macrophage supernatant did not show significant differences in activity.

## Discussion

We previously demonstrated anti-inflammatory and anti-fibrotic effects of GSK-3 inhibition in a mouse model of BLM-induced pulmonary fibrosis (Gurrieri et al., 2010). In the present study, we further investigated the role of GSK-3 in the early phase of ECM remodeling, which is known to play a pivotal role in IPF, focusing upon the modulation of MMPs and TIMPs that are essential in the physiological turnover of the matrix and in the repair of the disrupted BM.

Our *in vivo* studies used the BLM-induced mouse model of lung inflammation and fibrosis that, with all known limitations, is still the most used *in vivo* approach for studying IPF pathogenesis as well as potential anti-fibrotic drugs before phase I clinical trials (Scotton et al., 2013; Craig et al., 2015).

We found that MMP-2, MMP-9, TIMP-1, and TIMP-2 levels were increased in the BALF of BLM-treated mice, and we provided evidence that iAMs are, at least in part, responsible for the release of these mediators. We also demonstrated that SB216763-mediated GSK-3 inhibition strongly decreased MMP-9 activity and, to a lower extent, MMP-2 activity in the BALF of BLM-treated mice. Moreover, SB216763 significantly reduced MMP-9, MMP-2, TIMP-1, and TIMP-2 production in iAMs and cuboidalized type II epithelial alveolar cells at day 7. Consistent with our findings, it has previously been demonstrated in studies of dendritic spinal plasticity that increasing GSK-3β activity increases MMP-9 activity and that the non-specific GSK-3β inhibitor lithium is effective in down-regulating this metalloprotease (Kondratiuk et al., 2017).

Current understanding of the role of MMPs in IPF derives from expression levels in blood, BALF, and lung samples of patients with IPF and from mouse studies of MMP gene–targeted mice (Craig et al., 2015). Betsuyaku et al. found that fibrosing alveolitis develops in MMP-9–deficient mice after intratracheal bleomycin, irrespective of MMP-9 (Betsuyaku et al., 2000). However, MMP-9 facilitates migration of Clara cells into the regions of alveolar injury, thus favoring alveolar bronchiolization. To the same extent, patients with IPF showed increased production of matrix metalloproteinase-8 and -9 in the airways without a compensatory increase in TIMPs, suggesting that enhanced activity of MMPs may contribute to matrix disruption and remodeling in the development of fibrosis. Moreover, the *in vivo* use of the MMP inhibitor batimastat has been shown to inhibit MMPs, thus preventing BLM-induced pulmonary fibrosis (Corbel et al., 2001).

The role of MMP-2 in IPF pathogenesis is less defined. MMP-2 expression is increased in IPF lungs mainly in reactive airway epithelial cells and myofibroblasts (Fukuda et al., 1998). Of importance, by inducing targeted proteolysis of the BM, MMP-2 has been shown to promote EMT, with loss of epithelial features and acquisition of a mesenchymal phenotype.

We observed an increased expression of TIMPs following inflammatory and pro-fibrotic stimuli in our model. Although in different models, there is evidence that TIMP-1 and TIMP-2 expressions are increased within inflammatory environments. Interestingly, while induction of lung fibrosis in mice lacking TIMPs did not differ from that in wild type mice, the presence of TIMP-1 in mice has been shown to reduce inflammation, suggesting that TIMP-1 has a key role in restricting inflammation following lung injury (Kim et al., 2005; Manoury et al., 2006). The increased expression of TIMP-2 in bleomycin-induced pulmonary fibrosis has also been reported. Yaguchi et al. found immunoreactivity for TIMP-2 in bronchial and bronchiolar epithelial cells and in type II alveolar epithelial cells and alveolar macrophages (Yaguchi et al., 1998) after bleomycin treatment. Several studies demonstrated a role for TIMP-2 as a direct inhibitor of ECM proteolysis but also indirectly controlling ECM abundance, in some cases through activation of MMP-2 (Arpino et al., 2015). Moreover, TIMP-2 is unique in that it functions as both an MMP inhibitor and activator (Hernandez-Barrantes et al., 2000). It should also be taken into consideration that the balance between TIMPs and MMPs observed close to the inflammatory stimuli may change in the long term, therefore affecting ECM deposition.

Our BALF data combined with IHC results show that GSK-3 inhibition is effective in modulation of MMP-2 and MMP-9 and that macrophages and cuboidalized epithelial alveolar cells might act as the main characters of the play. However, whether SB216763 directly modulates MMP or acts indirectly through an anti-inflammatory effect cannot be addressed in the mouse model. To address this question and further understand the molecular mechanisms underlying the role of GSK-3 in MMP modulation, we then performed *in vitro* studies. There is evidence that MMP-9 can be activated directly from MMP-2 and that its expression can be modulated by GSK-3 downstream of c-Myc in oral squamous carcinoma cells (Pramanik et al., 2018). It has also been shown that inhibition of GSK-3 down-regulates the expression of MMP-2 and MT1-MMP in glioblastoma cells and that MMP-2 activation is mediated by the interaction of its pro-form with another metalloprotease, MT1-MMP, and TIMP-2 (Hernandez-Barrantes et al., 2000; Chikano et al., 2015). Surprisingly, in the A549 epithelial cell line, we found discrepancies between *in vitro* and *in vivo* effects of GSK-3 inhibition on MMP modulation following pro-inflammatory and pro-fibrotic stimulation. In fact, we could not confirm the strong reduction in MMP expression observed in mice after SB21 treatment as GSK-3 inhibition did not affect MMP protein expression in the A549 cell line. This is likely due to a limitation of our study, since we had no chance to use primary epithelial alveolar type II cells and we only tested our hypothesis using the A549 epithelial cell line that is derived from non-small-cell lung cancer tissue, a type of cancer where GSK-3 is known to be activated and involved in neoplastic proliferation and has been recently suggested as a potential therapeutic target (O'Flaherty et al., 2019; Xie et al., 2018).

Moving to lung fibroblasts, we then identified that MRC5 and primary IPF cells behaved differently in terms of MMP modulation. Interestingly, although the activity of MMP-9 in MRC5 cells was not significantly increased by TGFβ, SB216763 pre-treatment led to levels of MMP-9 secretion significantly lower than those in the basal condition, when combined with TGFβ stimulation, thus suggesting a specific effect on the TGFβ-induced cascade.

At the same time, TGFβ-induced pro-MMP-2 activity also decreased in MRC5 cells after pre-treatment with SB216763, but no significant modification has been observed in IPF cells under the same conditions. It is important to underline that, in our *in vitro* studies using MRC5 and primary IPF, we were unable to detect the active form of both MMP-2 and MMP-9 by zymography, as reported by other authors (Habelhah et al., 1999; Hostettler et al., 2014). Therefore, we acknowledge that measuring pro-MMPs is not reflecting their bioactivity that requires the interaction with other cell types.

Additionally, we demonstrated that GSK-3 inhibition significantly decreases αSMA protein levels in primary human IPF lung fibroblasts, upon TGFβ stimulation. The same effect was not observed in MRC5 lung fibroblasts. The increase in αSMA protein expression by fibroblasts is a well-known marker of fibroblast-to-myofibroblast (F–MF) transition. The different behavior of MRC5 and IPF fibroblasts in both MMPs and TIMPs and αSMA regulation under GSK-3 inhibition might reflect the relevance of microenvironment-related cell commitment, with IPF fibroblasts being *ex vivo* cells derived from fibrotic lungs.

Myofibroblasts are fundamental in restoring tissue integrity after wound healing by regulating the normal fibrotic process. However, myofibroblasts’ sustained presence stimulates dysfunctional repair mechanisms, causing excess contraction, extracellular matrix secretion, and, thus, fibrosis (Klingberg et al., 2013).

Our results confirm the potential role of GSK-3 inhibition in preventing fibroblast-to-myofibroblast transition upon TGFβ stimulation, particularly in IPF primary fibroblasts. This is in agreement with previous data demonstrating an effect of GSK-3 inhibition in decreasing αSMA protein levels in primary human lung fibroblasts, mediated by CREB phosphorylation (Baarsma et al., 2013). On the contrary, other researchers highlighted an opposite effect of GSK-3 inhibition on F–MF transition, mediated by the β-catenin pathway (Caraci et al., 2008). This is likely due to differences in cell types, adopted concentrations of TGFβ, and different ways of GSK-3.

According to our results, GSK-3 inhibition may thus induce an anti-fibrotic effect, by preventing F–MF transition, as suggested by the decrease in αSMA expression. Whether the effect of GSK inhibition on MMP and TIMP modulation, in this context, depends on direct regulation or simply on reduction in myofibroblast differentiation still needs to be investigated.

Our results from primary macrophages confirm that alveolar macrophages might also be a relevant target for GSK-3 inhibition, as suggested by IHC analysis of the mouse model. Macrophages are indeed sensitive to both the pro-inflammatory (TNFα) and the pro-fibrotic (TGFβ) stimulation, overlapping at day 7 after bleomycin-induced lung damage, and GSK-3 pharmacologic inhibition impacts on MMP-2 expression upon both stimuli. *In vitro* modeling of alveolar macrophages’ behavior, however, is not adequately representative of what is happening in the lung, since the microenvironment is crucial for such a “social” cell (Arora et al., 2018).

Due to their role in wound healing, the implication of macrophages and their environmental modulatory function in the pathogenesis of pulmonary fibrosis is still under investigation (Zhang et al., 2018).

Finally, our *in vivo* results may be relevant when considering whether inhibition rather than complete silencing of GSK-3 would be the optimal pharmacologic strategy. GSK-3 is a pleiotropic kinase implicated in many different pathways. It has been shown that GSK-3β knockout mice are embryonically lethal (Hoeflich et al., 2000). On the contrary, the long clinical experience with lithium teaches us that *in vivo* inhibition of this kinase is safe and effective. This is likely due to the partial inhibition that lithium exerts on GSK-3, which may be optimal for dampening GSK-3’s self-activating mechanisms in pathologic processes while allowing GSK-3 to exert, unhampered, its many other cellular actions (Beurel et al., 2015). Thus, it is not surprising that *in vitro* single cell culture experiments provide more uncertainties and discrepancies than *in vivo* results, suggesting that a more complex environment should be generated to obtain trustable *in vitro* results. This is further testified by the different behaviors highlighted between the MRC5 fibroblast cell line and primary IPF fibroblasts under the same culture and stimulation conditions, likely due to the pathologic context from which primary cells had been isolated bearing a sort of “environmental signature.” With this consideration, the pharmacologic *in vivo* inhibition performed in our mouse model might be not only closer to the possible application in clinical practice but also more suitable than *in vitro* experiments to really assess the role of the kinase and the actual potential of its inhibition. In addition to the potential benefit of only partial inhibition of GSK-3, in terms of future applications, the development of the disease-selective inhibition strategy of GSK-3 will hopefully be possible, based on the awareness of the specific mechanisms that regulate GSK-3 and that depend on GSK-3 in the specific pathologic context.

In conclusion, our *in vivo* studies showed that GSK-3 inhibition modulates MMP-2 and -9 and TIMP-1 and -2 expressions as well as activity in BALF and lung tissues, thus potentially limiting BLM-induced lung damage. Our *in vitro* experiments at least partially confirmed the effect of GSK-3 inhibition on macrophages and on fibroblasts, where SB216763 showed an impact on the expression of αSMA, a marker of fibroblast-to-myofibroblast transition. These results provide further hints about the role of GSK-3 in the pathogenesis of pulmonary fibrosis, a role still far from being clarified. Due to its implications at different levels in so many pathways involved in the development of fibrosis, GSK-3 remains a fascinating target in the field of IPF, where the aim of the research is not to close a bad way but to disrupt a dangerous network.

## Data Availability

The raw data supporting the conclusions of this article will be made available by the authors, without undue reservation.
