# Supplementary figures and images for: GSK-3 Inhibition Modulates Metalloproteases in a Model of Lung Inflammation and Fibrosis
Source: Front Mol Biosci. 2021 Jun 21;8:633054. doi: 10.3389/fmolb.2021.633054 (PMC8255387; doi:10.3389/fmolb.2021.633054)

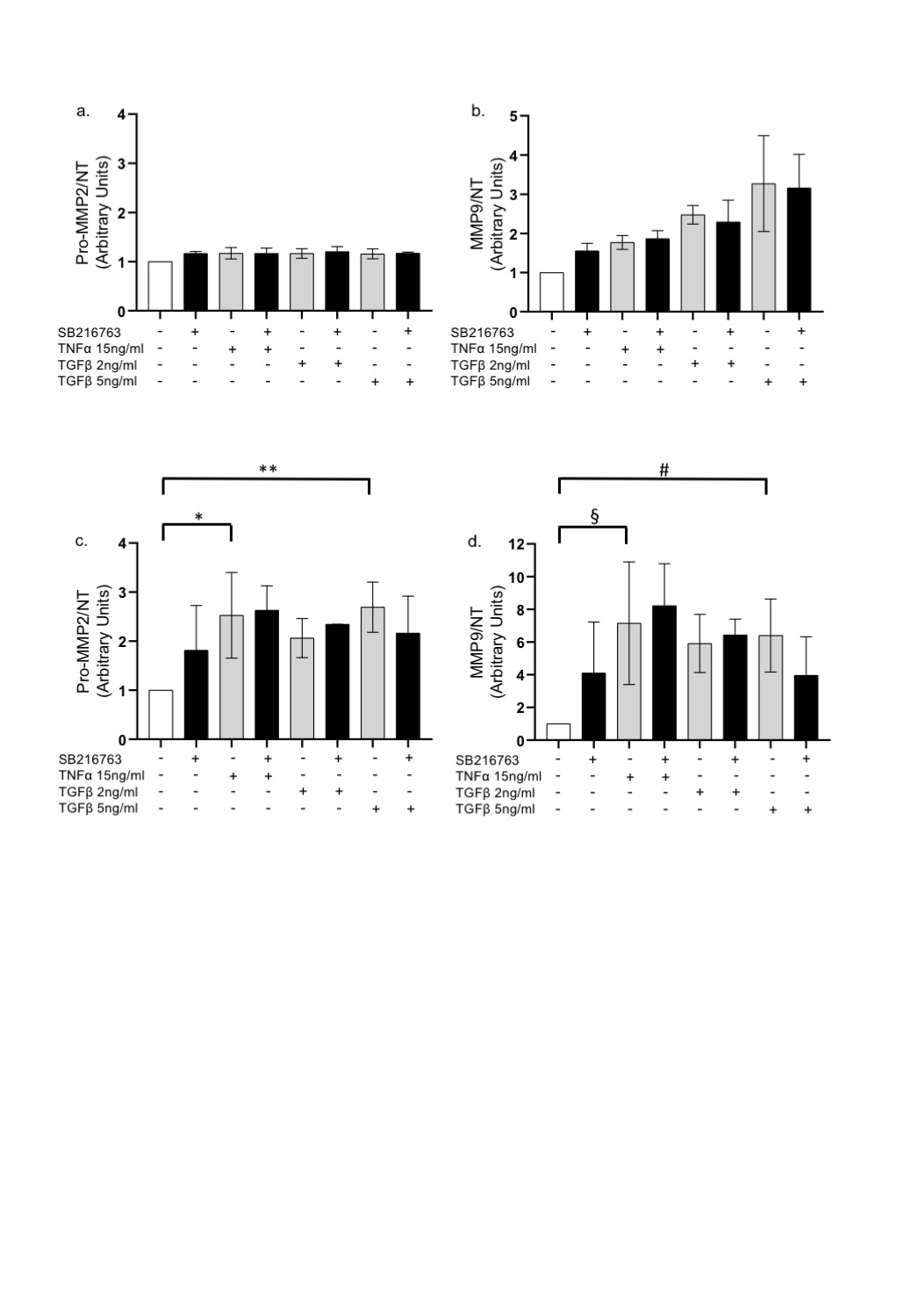

Supplement: Supplementary file 1 [file Image3.JPEG]

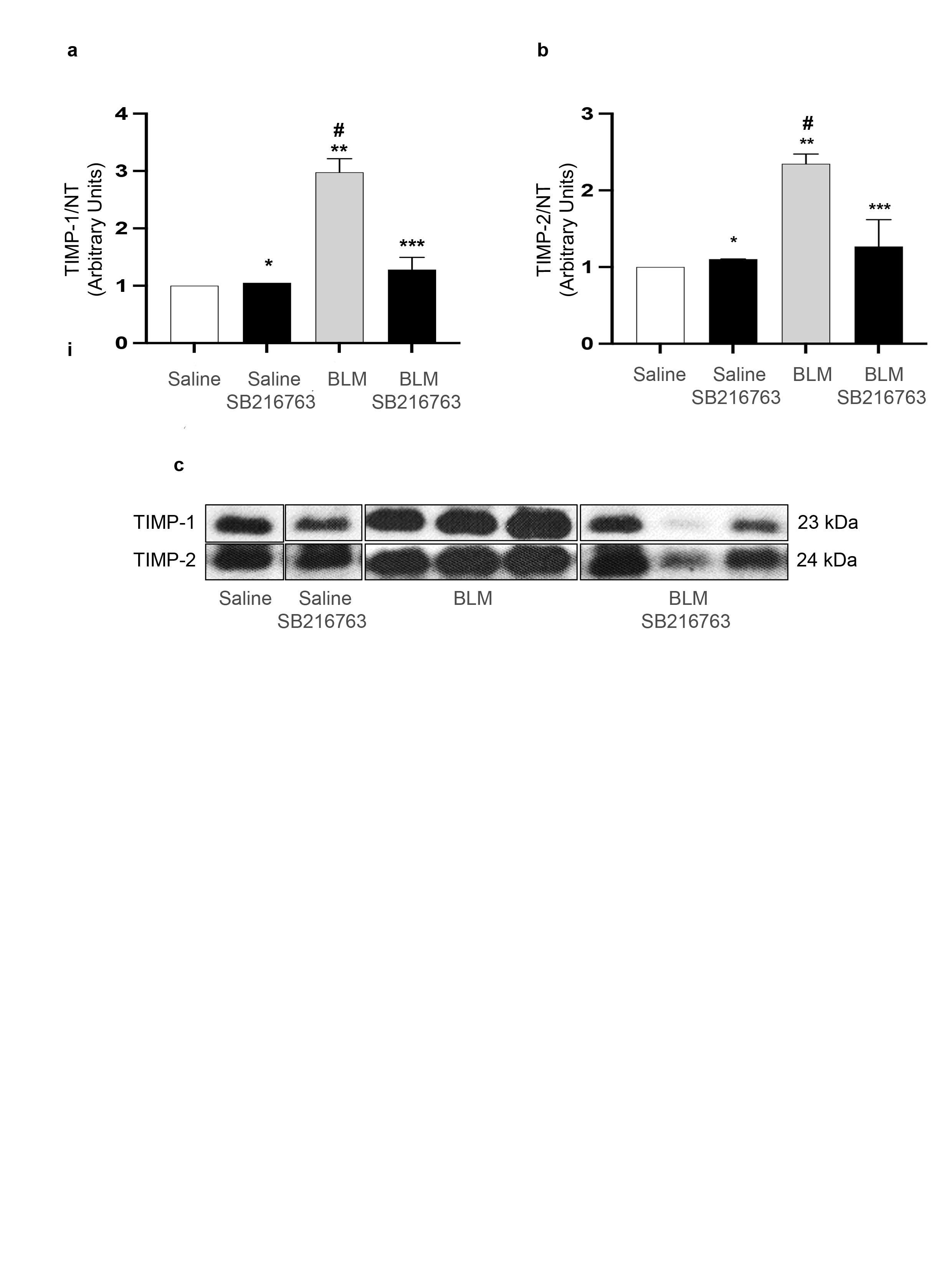

Supplement: Supplementary file 3 [file Image1.JPEG]

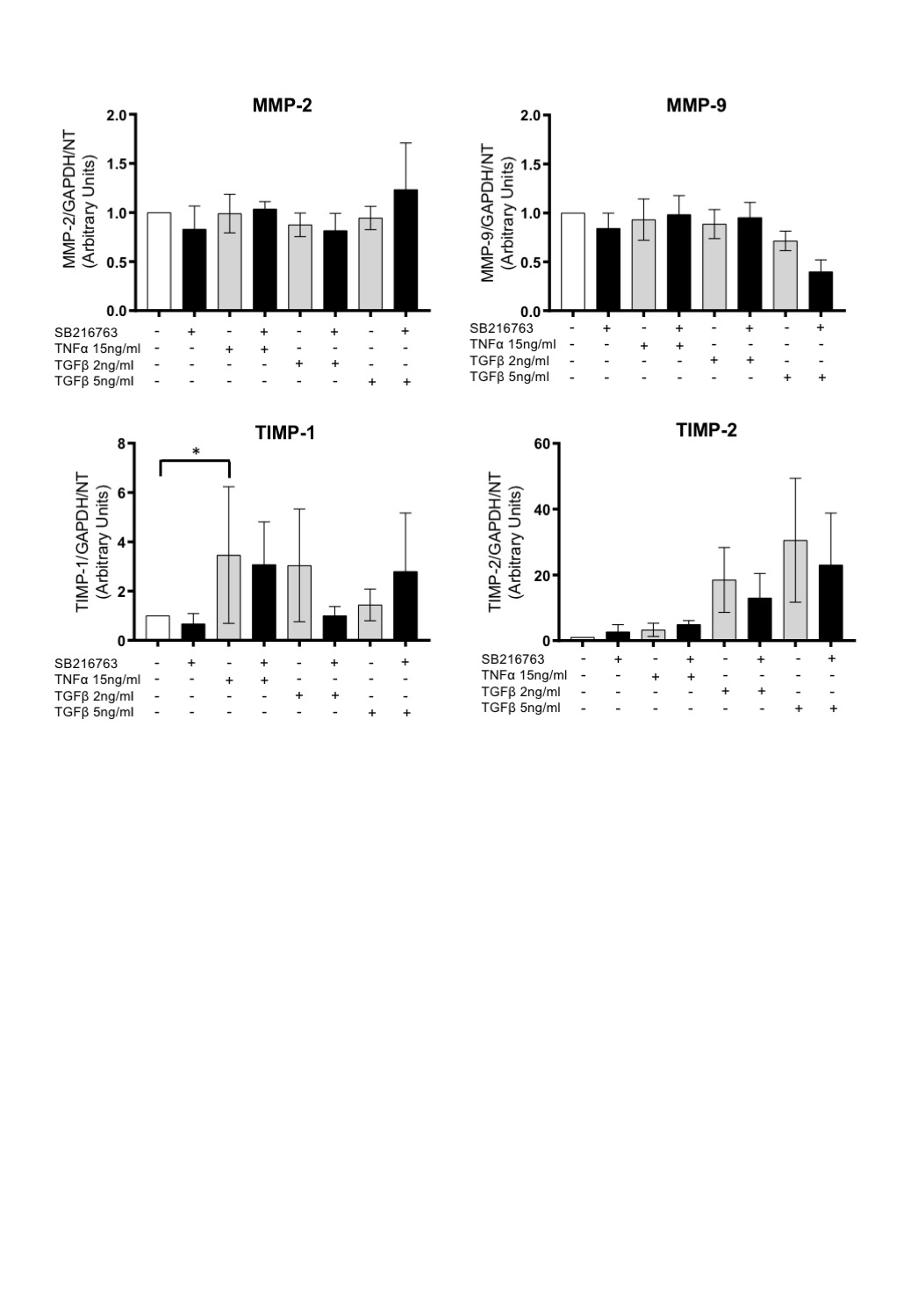

Supplement: Supplementary file 4 [file Image2.JPEG]
