# Supplementary material for: GSK-3 Inhibition Modulates Metalloproteases in a Model of Lung Inflammation and Fibrosis
Source: Front Mol Biosci. 2021 Jun 21;8:633054. doi: 10.3389/fmolb.2021.633054 (PMC8255387; doi:10.3389/fmolb.2021.633054)
Supplement: Supplementary file 2 [file Table1.DOCX]

| **Experimental**  **groups** |  | **total cells**  **(x 10^6^)** | **macrophages**  **(%)** | **lymphocytes**  **(%)** | **neutrophils**  **(%)** | **eosinophils**  **(%)** |
| --- | --- | --- | --- | --- | --- | --- |
| Saline  BLM  BLM + SB216763 | n=5/experiment  n=5/experiment  n=5/experiment | 1.7 ± 0.8  7.9 ± 4.3^Ψ^  2.6 ± 1.8* | 96 ± 1.2  62 ± 4.8  87 ± 6.4** | 4 ± 3.2  26 ± 7.0  11 ± 6.1* | -  10 ± 6.3  2 ± 1.5 | -  2 ± 1.5  - |

**Supplementary table 1.**

We performed total cell count of BALF recovered at day + 7 from mice of each experimental group. Total cell number significantly increased in BLM-instilled mice compared to control mice instilled with saline (p < 0.05). Moreover, the co-treatment of BLM-instilled mice with SB216763 significantly reduced total cell number of the BALF (p < 0.05). When evaluating BALF cell composition, macrophages represented the main cell population in control mice, as expected; in contrast, mice exposed to BLM showed a strong increase of lymphocyte percentage as well as the detectability of the two sub-populations of neutrophils and eosinophils. The inhibition of GSK-3 with SB216763 significantly reduced the lymphocyte percentage (p < 0.01), with a recovery of the physiological percentage of macrophages. This finding was in accordance with our previously published data of flow cytometric analysis, showing how the inhibition of GSK-3 induced a reduction in CD3^+^ T lymphocyte percentage at day + 7. (Gurrieri et al., 2010) An observed reduction of neutrophil and eosinophil percentage was not statistically significant.
